# Error in Nonauthor Contributors

**DOI:** 10.1001/jamanetworkopen.2023.5344

**Published:** 2023-03-10

In the Consensus Statement titled “Development of an Enhanced Recovery After Surgery Surgical Safety Checklist Through a Modified Delphi Process,”^1^ published February 8, 2023, there were typographical errors in the names of 2 nonauthor collaborators in the Additional Contributions and in Supplement 2. These should have appeared as Hans D. de Boer and Kevin Elias. This article has been corrected.^1^
